# Validating and Refining EPA’s Traffic Exposure Screening Measure

**DOI:** 10.3390/ijerph16010003

**Published:** 2018-12-20

**Authors:** Dana Rowangould, Greg Rowangould, Elena Craft, Deb Niemeier

**Affiliations:** 1 Sustainable Systems Research, LLC, Albuquerque, NM 87110, USA; dana@sustainablesystemsresearch.net; 2 Department of Civil and Environmental Engineering, University of New Mexico, Albuquerque, NM 87131, USA; rowangould@unm.edu; 3 Environmental Defense Fund, Austin, TX 78701, USA; ecraft@edf.org; 4 Department of Civil and Environmental Engineering, University of California, Davis, CA 95616, USA

**Keywords:** air pollution, traffic, EJScreen, spatial, roadway

## Abstract

Exposure to high air pollutant concentrations results in significant health risks. Many communities of color and low-income communities face disproportionately higher levels of air pollution exposure. Environmental justice (EJ) screening tools play a critical role in focusing early attention on areas with a high likelihood of disparate health impacts. In 2015, the United States Environmental Protection Agency (US EPA) released EJScreen, a screening tool with indicators of a range of pollution burdens across the US. However, little is known about the accuracy of the screening estimates of pollution exposure. This study compares EJScreen’s traffic proximity air quality metric to dispersion modeling results. Using the area around the Houston Ship Channel, we conduct fine-grained air pollution dispersion modeling to evaluate how closely EJScreen’s indicator approximates estimated roadway air pollution concentrations. We find low correlation between modeled concentrations and the EJScreen roadway air pollution indicator. We extend EJScreen’s roadway air pollution screening method in three ways: (1) using a smaller unit of analysis, (2) accounting for the length of each road segment, and (3) accounting for wind direction. Using the Houston region, we use two of the methods and show that the proposed extensions provide a more accurate transportation air pollution screening assessment at the regional and local level.

## 1. Introduction

Exposure to high air pollutant concentrations results in significant health risks, including increased cancer risks and adverse cardiopulmonary outcomes [1,2,3,4,5]. Many of these risks disproportionately burden communities of color and low-income communities [6], at the same time that these communities face greater vulnerability to air pollution exposure [7,8]. In light of these disparities, there are a number of legal and regulatory protections for people of color and low-income households in the United States (US) (including Title VI of the 1964 Civil Rights Act and Executive Order 12898, which has spurred agency guidance for evaluating environmental justice outcomes of agency decisions). There is also a legal framework supporting health effect assessments as part of the environmental review processes [9]. 

However, it may not be necessary (or feasible) to conduct comprehensive quantitative health assessments of air pollution exposure in every situation (e.g., in areas where residents are neither disadvantaged nor exposed to pollution). Environmental justice (EJ) screening tools (also referred to as cumulative impact and/or social vulnerability tools, e.g., [10]) play a critical role in focusing *early* attention on areas with a high likelihood of disparate health impacts due to environmental exposures. EJ screening tools can highlight areas where disadvantaged populations face high pollution levels and/or many pollution sources, providing decision makers and communities with insights that can help to focus subsequent attention [11]. EJ screening tools typically characterize the risk of disproportionate health burden in terms of proximity to various air pollutant sources or coarsely estimated concentrations or health risks (e.g., [10,11,12,13]). These environmental and health risk indicators are then combined with socioeconomic data (reflecting vulnerability) to identify areas where there is a high potential for elevated health risks and vulnerability. The metrics that are included in different tools vary, in part reflecting the differences in objectives as well as data availability and quality. For example, California’s screening tool, CalEnviroScreen, includes rates of low birth weight, asthma, and unemployment in its vulnerability measure, whereas United States Environmental Protection Agency’s (US EPA’s) screening tool does not [11,12].

For several years, the US EPA has employed screening tools internally and in 2010 they began developing EJScreen, a national environmental justice screening tool, incorporating insights from stakeholders and experts as well as from earlier research and screening tools such as CalEnviroScreen [11]. EJScreen was subsequently released to the public in 2015 [11]. EJScreen includes twelve environmental indicators (representing risks from a variety of air pollution, water, and hazardous waste exposures) and two demographic indicators (which are derived from up to six demographic measures) [11]. EJScreen data and maps present the environmental, demographic, and combined measures at the block group and census tract level to allow users to identify vulnerable communities which may experience high pollutant burdens. Importantly, the tool facilitates comparisons across communities, regions, and the nation without significant data gathering costs. The screening tool can be used by stakeholders and decision makers to identify areas where additional assessment or outreach may be merited [11]. In addition, because the tool is web-based, it allows for communities to access environmental and vulnerability data directly [11]. Providing access to analytical tools has the potential to empower communities to participate more substantively in decision-making processes [14,15]. This is important because many affected communities lack political and technical resources to advocate for effective mitigation strategies [16].

One of the EPA’s stated goals is “to ensure the screening tool [EJScreen] reflects an appropriate balance between simple, feasible, screening-level information on the one hand, and high-quality data and strong science on the other” [11] (page 8). Ensuring that screening tools are accurate predictors of more spatially resolved areas of high pollutant burden requires two things: accurate estimates of pollution sources (e.g., emissions locations and rates) and accurate methods for estimating pollution burdens (e.g., concentrations). For the former, Sadd et al. have examined the accuracy of stationary pollution source locations, finding that errors were substantial and that ground-truthing can change the predicted burdens reflected in screening methods [17]. For the latter, there has not been published research that has determined the degree with which screening tools can accurately identify the spatial distributions of high pollutant burdens. That is, how well do environmental justice screening measures reflect more detailed and reliable pollution estimates? While some amount of noise is inherent in the design of ‘screening’ tools, which are expected to be quickly deployed and serve as signals to warrant additional attention, comparisons to actual environmental burdens can reveal simple modifications that can improve accuracy and still be deployed at a ‘screening’ level of effort.

In this study, we explore the accuracy of one environmental measure included in EJScreen: the measure of roadway air pollution. This research is, to the best of our knowledge, the first study to examine the accuracy of a screening method’s estimation of pollution burdens by validating it using detailed modeling. Our analysis focuses on the area around the Houston Ship Channel. The Houston Ship Channel area contains many industrial and mobile air pollution sources, elevated air pollution concentrations [18,19,20], and is an area with a large share of low income people and people of color, all of which contribute to significant environmental justice concerns [21]. We use fine-grained air pollution dispersion modeling of four pollutants (similar to what is used when performing transportation air pollution conformity analysis) to evaluate the accuracy of EJScreen’s indicator of roadway air pollution concentrations. We then refine EJScreen’s roadway air pollution screening method in three ways: (1) using a smaller unit of analysis, (2) accounting for the length of each road segment, and (3) accounting for wind direction. Each of these refinements provides vehicle emissions concentration indicators that are more closely correlated with modeled vehicle emission concentrations than estimates that were produced using the EJScreen method. Finally, we demonstrate the application of two of the methods by identifying areas of environmental justice concern in the Houston region.

## 2. Methods

### 2.1. Study Area and Air Pollution Concentrations

**Figure 1:**
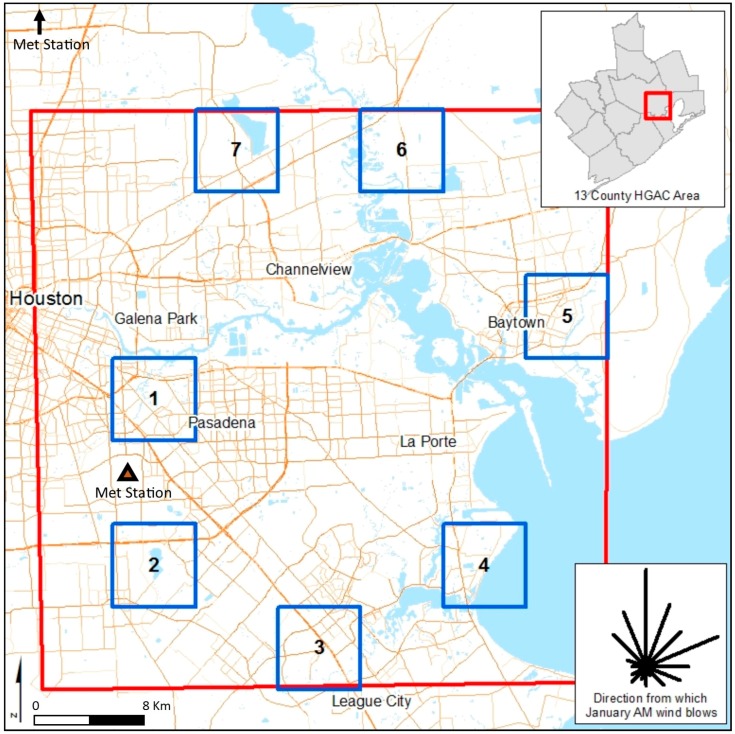
Seven sample areas around the Houston Ship Channel.

We selected seven areas from a 7 × 7 grid (49 squares), dividing a 1746 km^2^ area around the Houston Ship Channel (Figure 1); each sample area was approximately 36 km^2^. On-road vehicle emissions of volatile organic compounds (VOCs), total gaseous hydrocarbons (HC), and particulate matter (PM_10_ and PM_2.5_) were estimated for each roadway segment within one kilometer of each of the seven selected areas using US EPA’s Motor Vehicle Emission Simulator (MOVES) (2014a). MOVES is the required model for the regulatory modeling of mobile source pollutants. Traffic volume data were obtained from the Federal Highway Administration’s 2012 Highway Performance Monitoring System network [22]. Peak hour flow characteristics for the Houston area were used to generate morning peak hour traffic volumes; likewise, fleet characteristics were based on Houston data available through the Texas Department of Transportation (TxDOT). We also used MOVES default vehicle fleet age distribution and the Texas Commission on Environmental Quality’s (TCEQ) Meteorological Data for Refined Screening with AERMOD from two different meteorological stations in Harris County, Texas for temperature and humidity inputs [23].

US EPA’s AERMOD model (version 15181) was then used to estimate the concentration of vehicle emissions from each roadway segment. Houston has a long history of high pollutant levels with peaks in both the summer (ozone) and wintertime (air toxics). We modeled morning (8 to 9 a.m.) peak hour concentrations for the month of January, because we expect the tools to perform better when air pollutant levels are higher. AERMOD was set to run with five years of meteorological data processed with AERMET (version 12345) corresponding to the 8 a.m. hour for days in January (obtained from TCEQ) following the approach described in Rowangould [24] for modeling large transportation networks. Concentrations were estimated for a 100 m grid of receptors, which were then spatially interpolated using empirical Kriging in ArcGIS to a raster with a 20 m resolution. The vehicle emissions concentration rasters are used to calculate average concentrations for the 2116 populated US Census blocks with centroids located in one of the seven sample areas. We focus the remainder of this analysis on just PM_2.5_, because the concentration estimates for the four pollutants that we modeled are highly correlated for blocks in the sample areas (ranging from 0.92 to 0.99, see supplementary material) and because US EPA requires air dispersion modeling to evaluate near roadway PM_2.5_ concentrations for certain transportation projects in National Ambient Air Quality Standard non-attainment areas [25]. In our analysis, we are only evaluating air quality impacts from primary vehicle emissions along roadways, and not regional impacts from secondary pollutants, such as secondary organic aerosols (a large source of urban PM_2.5_) or ozone, which are also partially attributable to vehicle traffic. While each of the pollutants we evaluated have similar concentration patterns along roadways, how much these pollutants contribute to the overall air quality in a given are depends on the level of regional background concentration. 

In contrast to screening tools that use concentration and health risk indicators, dispersion modeling provides estimates of actual air pollution concentrations, taking into account the magnitude of emissions, topography, and meteorological conditions. Therefore, we treat these estimates as the true concentration of air pollutants for the purposes of our analysis. 

### 2.2. Roadway Air Pollution Screening Indices 

We use EPA’s EJScreen method to estimate a roadway air pollution index in the seven sample areas and across the Houston Galveston Area Council (HGAC) region. EJScreen’s method accounts for proximity to roads and traffic volume on each road. We also extend the EJScreen approach in three ways: deriving new roadway air pollution index estimates at the block level, accounting for the length of roadway segments, and accounting for wind direction (Table 1). We describe the calculations for EJScreen and each adjustment below.

#### 2.2.1. EJScreen

We follow the methods used in EJScreen to approximate the air pollution impacts of traffic volume and proximity. We use block-level 2010 US Census population data and 2012 Highway Performance Monitoring System (HPMS) roadway and traffic data to calculate the roadway air pollution index $P_{Ri}$ for the 137,577 US 2010 Census blocks within the 13 county HGAC area as

(1) $$P_{Ri}=\overset{J_{i}}{\underset{j=1}{\sum }}\frac{W_{ij}}{d_{ij}}$$

where $W_{ij}$ is the weight of the jth 2012 HPMS roadway segment and it is set equal to the 2012 HPMS annual daily traffic volume, estimated for $J_{i}$ mobile pollution source segments that are within 500 m of the ith block centroid (a distance of 500 m is often used to approximate the area of near-road air pollution impacts and is consistent with the method used in US EPA’s EJScreen). $d_{ij}$ is the shortest distance from the ith block centroid to the nearest part of the jth HPMS roadway segment. For block centroids that are very close to the road segment (where $A_{i}$ is the ith block’s area and $d_{ij}<0.9\sqrt{A_{i}/\pi }$) a corrected distance equal to $0.9\sqrt{A_{i}/\pi }$ is used instead of $d_{ij}$. This correction approximates an exposure distance for a uniformly distributed population assuming that the block is circular. (The criterion we applied to determine which centroids are very close to the road is consistent with the EJScreen Technical Documentation [11], although an examination of the EJScreen calculation code (obtained via personal communication with EPA staff) indicates that the actual criteria applied was $d_{ij}<\sqrt{A_{i}/\pi }$. In both the technical documentation and the code the corrected distance used for blocks identified as close to the road is $0.9\sqrt{A_{i}/\pi }$. We estimated our results using both methods (not shown) and found that the differences were minimal, because a small share of blocks have centroids between $0.9\sqrt{A_{i}/\pi }$ and $\sqrt{A_{i}/\pi }$). For block centroids with no HPMS roadway segments within 500 m, the nearest single HPMS road segment was used. The block level air pollution indices were then averaged at the block group level and weighted by the population within each block to arrive at a block-group level roadway air pollution index for 3108 block groups (all block groups with populated blocks that fall within 500 m from the edge of the 13 county HGAC area). For five block groups with no populated blocks, the average of all block level air pollution indices in the block group was used. The air pollution index was estimated for a total of 3113 block groups in the HGAC area. 

**Figure 2:**
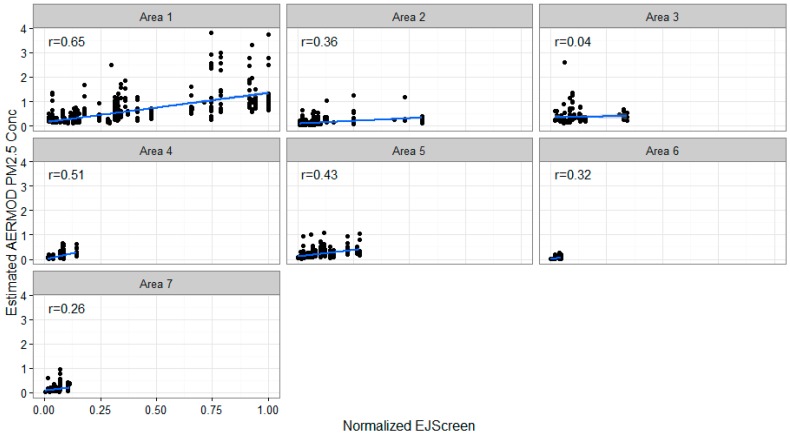
Particulate matter (PM_2.5_) and normalized EJScreen index values.

We compared the modeled PM_2.5_ concentrations and the normalized EJScreen roadway air pollution index values across the subset of 2116 populated census blocks with centroids that are located within the seven study areas shown earlier in Figure 1. We focus on populated census blocks, because many of the unpopulated census blocks follow roads and our analysis is intended to reflect impacts to residents (not on roadways). EJScreen estimates, which are derived at the block group level, are assigned to the corresponding blocks to allow for a block level comparison. The estimated correlations between the normalized EJScreen roadway air pollution index and the modeled PM_2.5_ concentrations (shown as µg/m^3^ from road sources only) were moderate to low: 0.65; 0.36; 0.04; 0.51; 0.43; 0.32; and 0.26 for areas 1 through 7, respectively (Figure 2). The lack of a strong relationship between EJScreen’s roadway air pollution index and the modeled roadway PM_2.5_ concentrations can be seen with the substantial scatter around each fitted line. Ideally, correlations between the modeled concentrations and the screening metric would be higher. The EJScreen roadway air pollution index in its current form is not accurate enough to predict block-level roadway pollutant burdens in many areas, and thereforeit may not be particularly helpful at (and may actually hinder) predicting higher risks at localized scales. (Note that our EJScreen air pollution index values differ from the comparable traffic intensity measure (PTRAF) in the US EPA’s EJScreen. US EPA’s 2015 EJScreen uses 2008 HPMS data while our analysis uses 2012 HPMS data. The 2008 HPMS data represents a much sparser highway network than 2012 HPMS data in the study area, reflecting only very busy roads.) We then considered three refinements that have the potential to improve the correlation between the modeled roadway pollution concentrations and the EJScreen roadway air pollution index.

#### 2.2.2. Block Level

The calculations here are the same as the EJScreen method, however, roadway air pollution indices are not aggregated to the block group level, so they remain disaggregated to census blocks. We derived this correction in order to evaluate estimates at a higher spatial resolution, which is expected to provide greater accuracy at a local scale. Values are estimated for 72,148 populated blocks of the 137,577 blocks in the HGAC region (excluding 65,265 unpopulated blocks and an additional 164 populated blocks with centroids that fall within 500 m of edge of the 13 county HGAC area).

#### 2.2.3. Length

**Figure 3:**
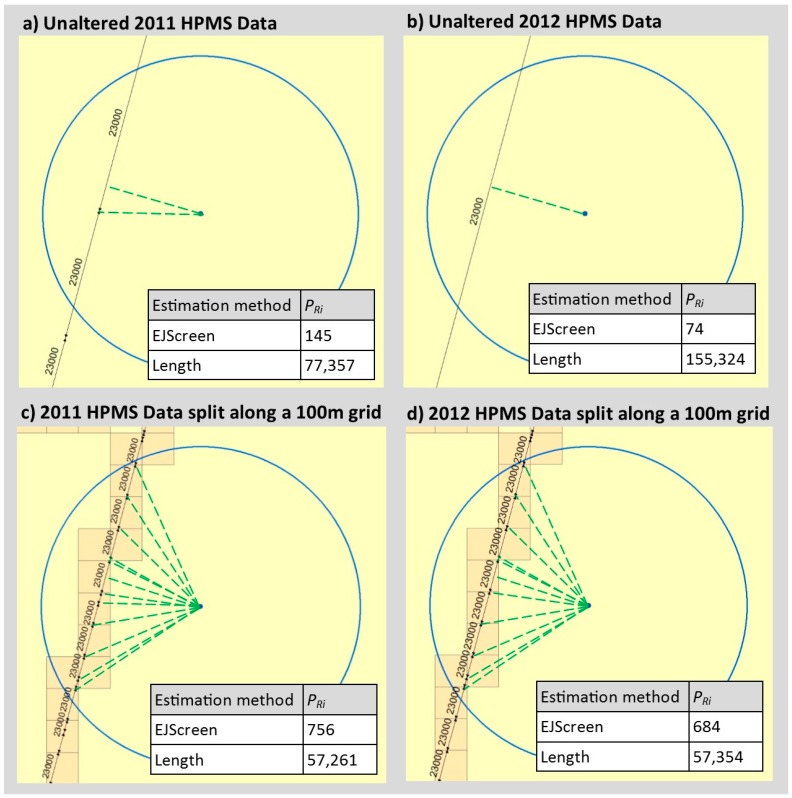
Block level roadway air pollution index calculations for an example census block (Block 482013139001052). The road segmentation varies in each panel but the traffic level is the same (23,000 annual average daily traffic). Each panel illustrates the block centroid, a 500 m circular buffer around the block centroid, nearby highway performance monitoring system (HPMS) road segments, dashed lines connecting the block centroid to the nearest part of each road segment, and the EJScreen and Length estimates for the block. Panel (**a**) shows unaltered 2011 HPMS data, panel (**b**) shows unaltered 2012 HPMS data (**c**) shows 2011 HPMS data split along a 100 m grid, and (**d**) shows 2012 HPMS data split along a 100 m grid.

Here, we applied a correction to the Block Level estimates to account for the varying lengths of the HPMS roadway segments. The 23,931 HPMS roadway links in the HGAC area range from 1.5 to 16,820 m, with an average length of 607 m. In the HPMS dataset, some segments are short, because traffic volumes change at frequent intervals along the length of the road, while others are divided into many shorter roadway segments, even as traffic volumes remain constant. In blocks where HPMS road segments are short, the EJScreen and Block Level methods will produce a higher roadway air pollution index than if the road were one long segment because they will count the same road multiple times. Figure 3 shows the roadway air pollution index values ($P_{Ri}$) for an example block, estimated using the EJScreen and Length methods for different versions of the road network as reflected in 2011 and 2012 HPMS data (segment endpoints differ but traffic volumes remain the same.) Comparing the EJScreen estimates of $P_{Ri}$ in Figure 3a and Figure 3b demonstrates the data artifact that arises due to variations in the segments of the HPMS network: the 2011 EJScreen estimate is nearly double the 2012 estimate due to a split in the road segment that occurs within 500 m of the block centroid.

We hypothesized that a length-based correction would reduce the influence of this data artifact and better correspond to estimated PM_2.5_ concentrations. Thus, we derived index estimates similarly to the Block Level analysis, except that $W_{ij}=v_{j}l_{j}$, where $v_{j}$ is the HPMS annual daily traffic volume and $l_{j}$ is the road segment length. However, this approach risks erroneously including portions of long segments that extend well beyond 500 m from a block centroid. For example, in Figure 3b, the included road segment has a length of over 2100 m, extending well beyond 500 m from the block centroid. To reduce the potential for erroneous inclusion of distant portions of roads, we first modified the HPMS network by dividing it along a 100 m square grid. This shortens the HPMS segments (with a new min/average/max of 0.002/73/194 m for 198,566 segments). These shorter road segments also provide a more granular estimate of the distance between each portion of the road and the block centroid. This modified network is used to derive $W_{ij}$. Comparing Figure 3c,d shows that differences in the road data’s segmentation (as shown in the 2011 to 2012 HPMS data) result in less than 0.2% change in the Length estimates when using this modified network.

#### 2.2.4. Wind

We applied an additional correction to the Length estimates to account for wind direction. We hypothesized that this correction might capture subtleties in the spatial distribution of emissions transport. We modified $W_{j}$ by adding a wind factor. For most road segments (located more than $0.9\sqrt{A_{i}/\pi }$ from the block centroid)

(2) $$W_{ij}=v_{j}l_{j}w_{ij}\;\mathrm{for}\;d_{ij}>0.9\sqrt{A_{i}/\pi }$$

where $w_{ij}$ is the percent of time that wind typically travels from the jth road segment toward the ith block centroid. We use January morning (8 to 9 a.m.) 1984 to 1992 hourly wind direction frequencies from the Houston International Airport obtained from the US EPA SCRAM surface meteorological archived data, which is also reflected in the AERMET data used for the dispersion modeling. The percent of time is calculated for each of 16 directions. For roadway segments that are located near a block centroid (within a radius of $0.9\sqrt{A_{i}/\pi }$), the wind factor that is used is $w_{calm}$, which is the percent of time in which there are calm conditions (without reference to the direction of the wind):

(3) $$W_{ij}=v_{j}l_{j}w_{calm}\;\mathrm{for}\;d_{ij}<0.9\sqrt{A_{i}/\pi }$$

## 3. Results and Discussion

**Figure 4:**
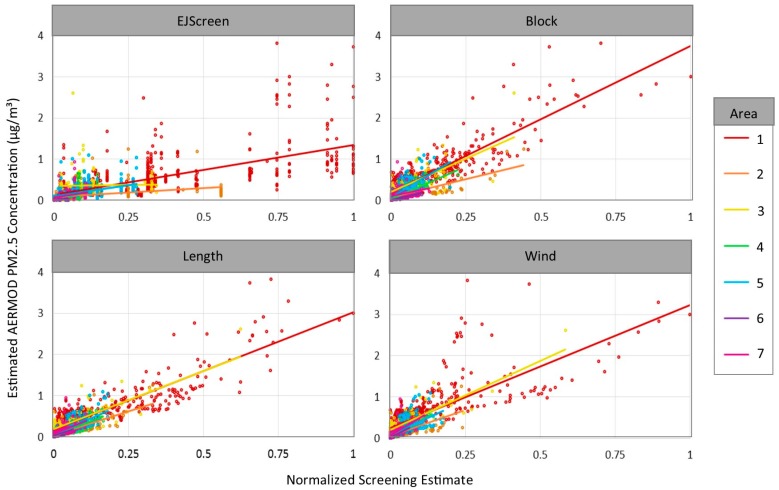
PM_2.5_ concentrations versus normalized roadway air pollution screening indices.

Each of our proposed refinements to the EJScreen method significantly improves the correlations between the modeled PM_2.5_ concentrations and the respective roadway air pollution indices for the 2116 populated blocks in the sample areas (Table 2, Figure 4). The correlations dramatically improve with the Block Level correction, and additional improvement is seen with the Length correction. Improvements are particularly dramatic in Area 3, where the EJScreen method diverges substantially from the modeled concentrations, which is likely due to variability in the location of highly populated blocks because the EJScreen block-group level estimates are a population weighted average of block-level estimates. The Wind correction does not produce a clear improvement over the Length correction.

In the Supplementary Materials, we compare the Length and Wind screening method percentiles and the modeled PM_2.5_ concentration percentiles along a number of dimensions. The relationship between each screening estimate and the modeled PM_2.5_ concentrations are similar. Both methods underestimate pollutants when the nearest road is between 500 and about 800 to 1000 m away, indicating that the 500 m cutoff for multiple road inclusion may be too small. For example, if roads at 800 m have an effect, then error would be introduced when only the nearest of several road segments located 600 m away are included in the assessment. There is, however, no clear reason for the lack of additional clarity brought by the Wind method. It is possible that additional refinements to the wind method (such as accounting for wind speed, which affects pollution distance, adjusting the distance pollution is assumed to be transported by “calm” wind conditions, or aggregating to fewer than 16 directions) would result in improved estimates.

**Figure 5:**
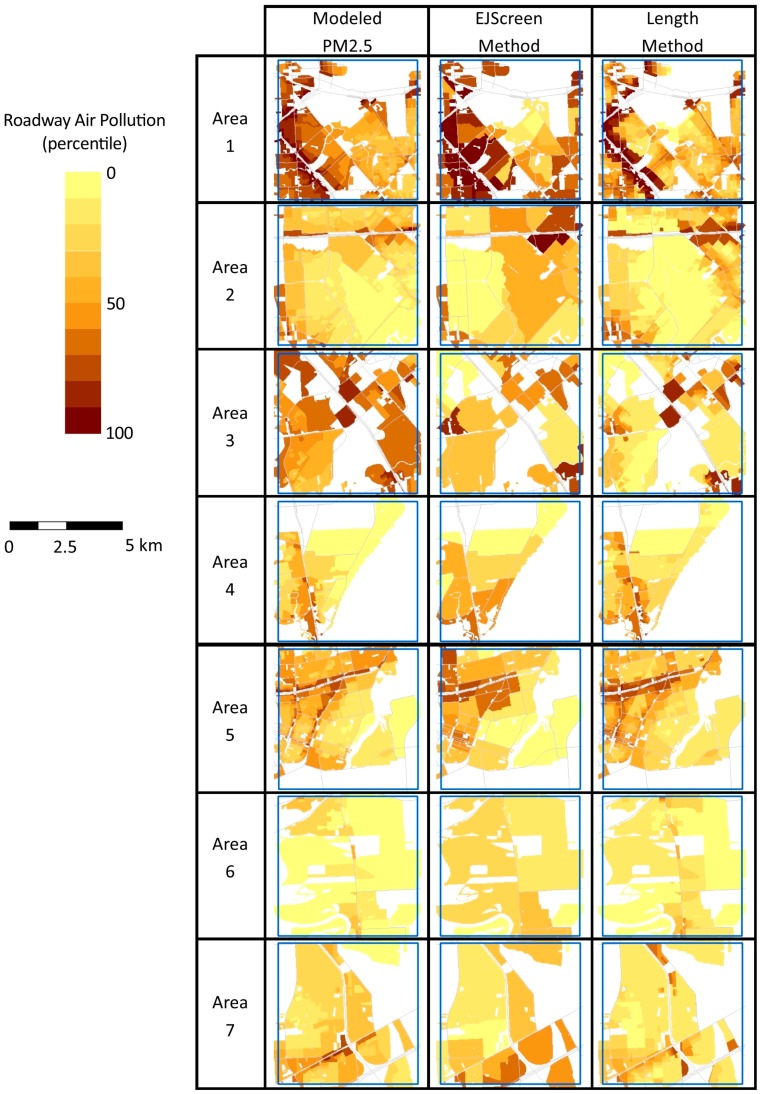
Comparison of modeled PM_2.5_ and roadway air pollution indices for populated census blocks using EJScreen and Length methods. The block group level EJScreen estimates are assigned to the corresponding populated census blocks. Percentiles are estimates relative to all sample areas combined.

Figure 5 maps the modeled PM_2.5_ concentration and the EJScreen and Length roadway air pollution indices for each of the seven sample areas. Metric values are shown as a percentile to facilitate visual comparisons. Percentiles are estimated relative to values in all sample areas combined, so that identical concentrations have the same percentile in different sample areas. As might be expected given the results that are shown in Table 2, the Length correction appears to better approximate modeled PM_2.5_ roadway concentrations in most areas. In contrast, the roadway index for EJScreen tends to show greater concentrations in locations that are further from roadways; this is largely due to the coarse grain of a block group-level analysis. In Figure 4, the Length method appears to have the weakest relationship to modeled pollutants in Area 3, although Table 2 and Figure 5 indicate that it performs reasonably well. This difference relates to the different sizes of the blocks; in Area 3, many small blocks perform well, while several large blocks perform poorly (but dominate the visual impression). Additionally, Areas 1 and 3 present visual examples of the underestimates exhibited by the Length method for blocks with centroids that are more than 500 m from a busy road (consistent with the discussion above).

To better understand how the new index translates into the identification of areas with the greatest potential for environmental disparities in the region, we also calculated a block-level vulnerability index as the average of the percent of residents that are people of color (non-white and/or Hispanic, using the 2010 Census) and the percent of residents that are low-income (living at less than twice the poverty level, from five-year 2013 American Community Survey). We then multiply the Length roadway air pollution index percentile by the vulnerability index for each block to derive a Length roadway EJ index. This approach is consistent with one approach that is used in EJScreen (Supplementary EJ Index 2 using the Demographic Index), except that the analysis is conducted at the block level. To derive an EJScreen roadway EJ index, we estimate the vulnerability index at the block group level and multiply it by the EJScreen roadway air pollution index (also derived at the block group level). We also translate both roadway EJ indices into percentiles (relative to the entire region) to facilitate comparisons.

**Figure 6:**
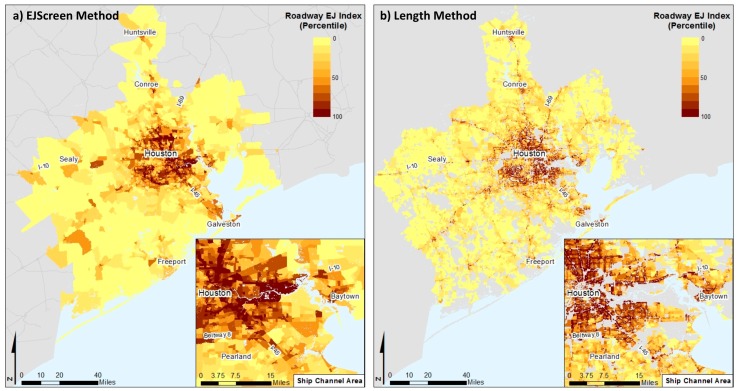
Comparison of Roadway Environmental Justice Indices using the EJScreen and Length Methods. Percentiles are estimated relative to the region.

We compared the differences in the areas of concern across the region identified by the two different roadway EJ indices. Figure 6 shows the roadway EJ index values as percentiles, where darker shades highlight areas that have both elevated roadway pollution burdens and elevated vulnerability. The roadway EJ index that is arrived at using the Length method captures finer grained areas and identifies areas near high traffic volume roads. In contrast, the highest EJ index values using the EJScreen method are large areas; these block groups are so large that much of the area is not adjacent to roads.

**Figure 7:**
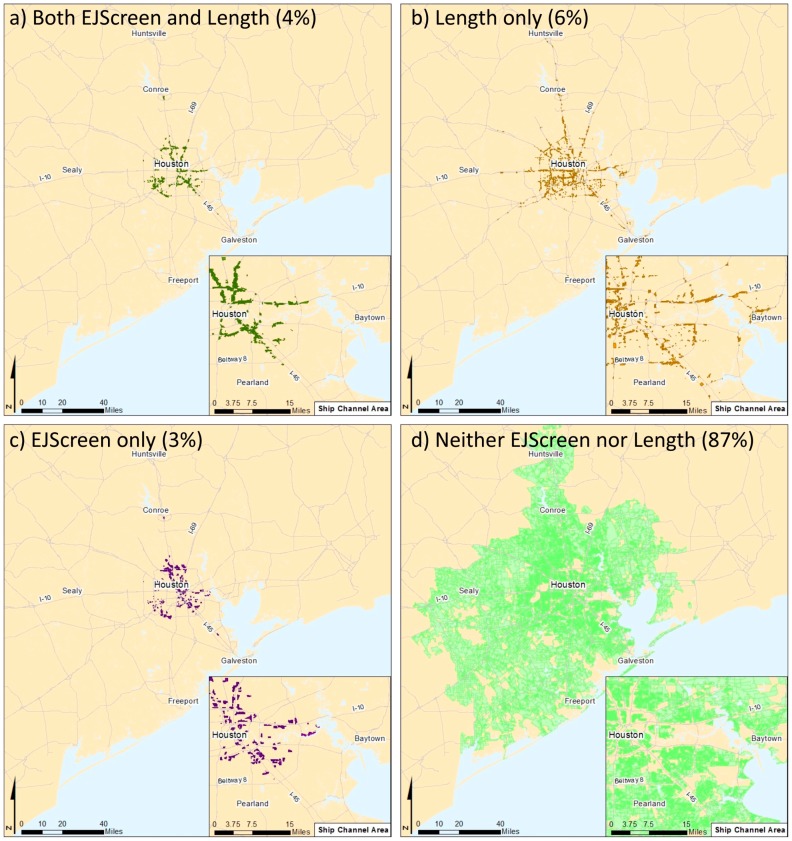
Comparison of areas where environmental justice concerns are elevated, identified as populated blocks with environmental justice (EJ) index values that are above the 90th percentile for the region. Areas of concern that are identified using both the EJScreen and Length methods are shown. Part (**a**) shows areas of elevated concern indicated by both methods, part (**b**) shows areas of elevated concern indicated by the Length method only, part (**c**) shows areas of elevated concern indicated by EJScreen only, and part (**d**) shows areas that are not identified using either method (populated blocks with EJ index values that are below the 90th percentile).

If we identify areas of concern as populated blocks that are at or above the 90th percentile for the EJScreen and Length roadway EJ indices (Figure 7), we find that both methods identify 4% of all blocks in the region (Figure 7a), 6% of blocks are identified by the Length method only (Figure 7b), 3% are identified by the EJScreen method only (Figure 7c), and 87% of the blocks are below the 90th percentile using both methods (Figure 7d). Figure 7 shows that there is likely a misclassification of environmental justice concerns, with the areas identified using each method differing more than they overlap. As discussed above, the Length method captures a finer grained area near busy roads. In light of the greater correspondence of the Length method to the modeled air pollution concentrations, we see that, at fine-grained local scales, EJScreen fails to capture many areas of concern.

## 4. Conclusions

We have demonstrated that the roadway air pollution index method used in EJScreen can be adapted to provide a more accurate transportation air pollution screening assessment at the regional and local level by applying the screen at the census block level and correcting for the roadway length. We note that these improvements do not require any additional data, as they rely on the same traffic data source that is currently used in EJScreen. With these refinements, EJScreen shows a stronger correlation with modeled roadway pollutant concentrations and better identifies potentially impacted environmental justice communities.

The refined index that is proposed here could be applied to the evaluation of transportation pollution reduction policies at regional and local scales. For example, the refined screening index might be used to conduct preliminary assessments of the impacts of truck idling reduction programs, clean fleet requirements, VMT reductions, and other programs that target a reduction in transportation emissions. Although our refinements of EJScreen were directed to the local and regional levels, it is possible that the new approach would also improve larger scale analyses, although the refinement may require more experimentation for larger networks. If these improvements were incorporated into future versions of EJScreen, then these more refined insights would also be readily available to decision makers and to disadvantaged communities.

There are several limitations to the refined index proposed here. The refinements to EJScreen are most suitable for analyzing potentially high roadway emissions. The ability of the refined screening tool to accurately reflect mobile source pollutant burdens will be a function of the spatial resolution of the roadway network. Future research might explore using a radius that is greater than 500 m, estimating the screening method for a fine grid (rather than at block centroids), and assigning the average of all included points to each block; improving the Wind method by accounting for the wind speed, adjusting the dispersion radius for calm conditions, or using fewer wind directions (or an average of several adjacent wind directions).

This analysis focuses on just one metric included in EJScreen: the roadway air pollution metric. Similar refinements could also be explored for other pollution metrics that are included in EJScreen or other screening tools using other data sources and/or methodological improvements. These refinements could use an approach that is similar to what is presented here, whereby refinements are validated using more detailed modeling of pollution burdens.

## Figures and Tables

**Table 1:** EJScreen method and three extensions.

Roadway Air Pollution Indices	Traffic Volume and Proximity	Block Level Analysis	Roadway Length Adjustment	Wind Direction Adjustment
EJScreen	Y	N	N	N
Block Level	Y	Y	N	N
Length	Y	Y	Y	N
Wind	Y	Y	Y	Y

**Table 2:** Correlation between PM_2.5_ and roadway air pollution screening index for populated blocks. Bold values indicate the highest correlations for each sample area.

Area	N	Correlation between PM_2.5_ and Screen Value			
		EJScreen	Block	Length	Wind
1	648	0.65	0.89	**0.89**	0.77
2	366	0.36	0.75	**0.80**	0.69
3	147	0.04	0.70	0.80	**0.83**
4	164	0.51	0.85	0.85	**0.90**
5	499	0.43	0.76	**0.77**	0.66
6	134	0.32	0.71	**0.74**	0.72
7	158	0.26	0.36	0.55	**0.64**

## Supplementary Materials

Supplementary Materials are available online at http://www.mdpi.com/1660-4601/16/1/3/s1. The Supplemental Materials provide data on the region so that information within study area can be contextualized. The SM also provides correlations of the four pollutant estimates and detailed comparisons of the Length and Wind screening estimates to the modeled PM_2.5_ concentrations.
