# Supplementary material for: Validating and Refining EPA’s Traffic Exposure Screening Measure
Source: Int J Environ Res Public Health. 2018 Dec 20;16(1):3. doi: 10.3390/ijerph16010003 (PMC6338909; doi:10.3390/ijerph16010003)
Supplement: Supplementary file 1 [file ijerph-16-00003-s001.pdf]

S.1. Correlation between dispersion modeling-derived concentrations of HC, VOC, PM2.5, and PM10 for 2,116 populated blocks

	PM2.5	PM10	HC
**PM10**	0.99		
**HC**	0.92	0.92	
**VOC**	0.92	0.92	1.00

S.2. Number of Blocks and Block Groups in each area

	Census Blocks		Census Block Groups	
	**All**	**Populated**	**All**	**Populated**
**HGAC region (entire)**	137,577	72,312	3,148	3,143
**HGAC region (centroid within 500 m from edge)**	137,084	72,148	3,113	3,108
**7 study areas (centroid within)**	4,114	2,116		

S.3. Examination of the differences between length and wind and the modeled PM2.5 concentrations.

We compare the Length and Wind screening estimates to the modeled PM2.5 concentrations. All estimates are converted to a percentile (with reference to the 2,116 populated blocks with centroids within the seven modeled areas).

Below we examine overall trends for all 2,116 blocks. There is little apparent difference between the Length and Wind estimates and their ability to match the modeled PM2.5 percentiles.

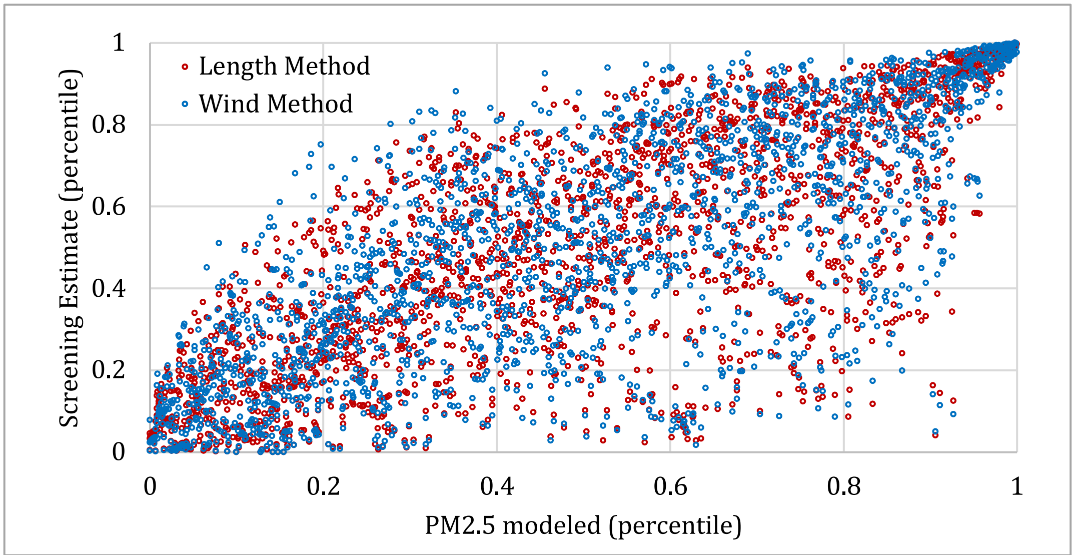

Below we examine the 2,116 blocks in terms of block size (shown on a logarithmic scale) and the difference between the Length / Wind estimates and the modeled PM2.5 percentiles. There is no clear relationship to between block size and the ability of the Length and Wind method to match modeled PM2.5 percentiles.

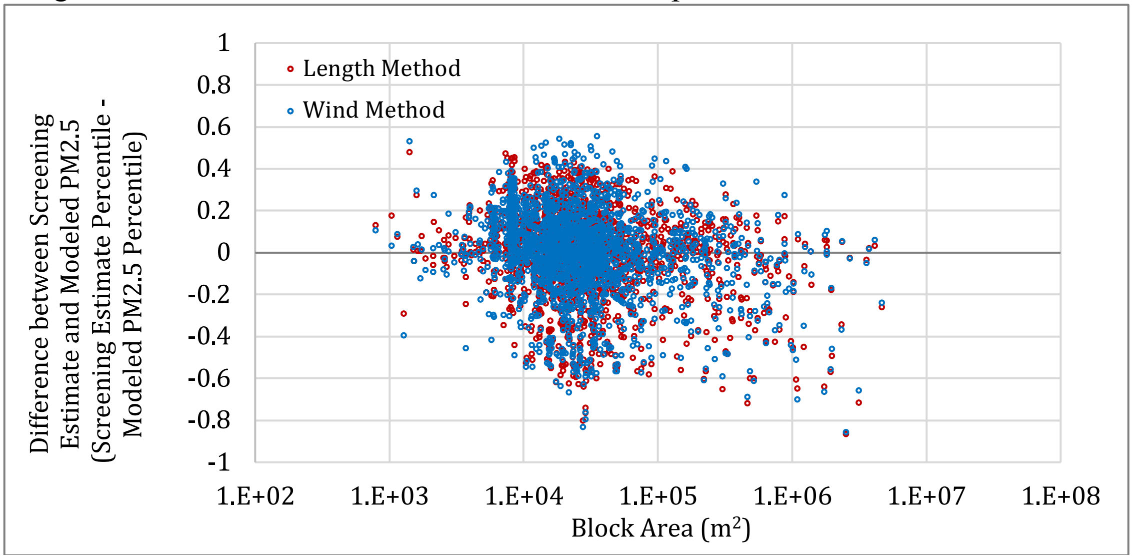

We then examine a subset of 176 of the 2,116 blocks that have screening estimates that are based on only one nearby road segment. This allows us to isolate a few factors that are particular to the road or its relationship to the block centroid. 175 blocks have screening estimates that are based on the nearest road segment that is greater than 500 meters away (one block has only one segment within 500 meters and it is located 499.9 meters away). Below we examine the relationship between the road segment length and the difference between the Length/Wind estimates and the modeled PM2.5 percentiles. While it looks as though the majority of these screening estimates are underestimated, there is no clear relationship between road segment length and the ability of the Length and Wind method to match modeled PM2.5 percentiles for this subset of blocks.

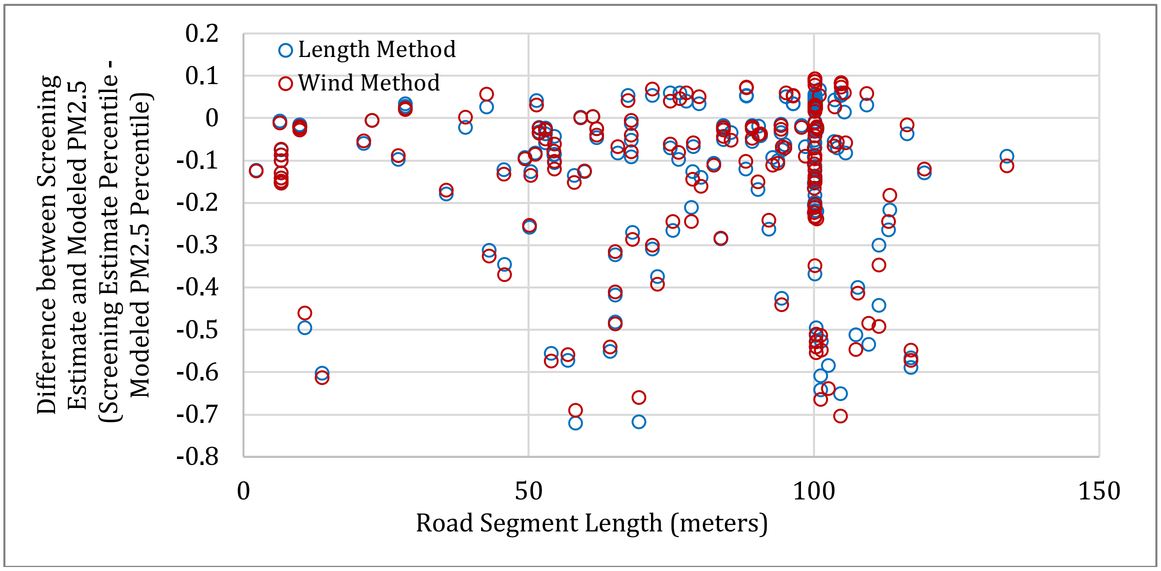

For the same subset of 176 blocks, we examine the relationship between the distance to the road segment and the difference between the Length/Wind estimates and the modeled PM2.5 percentiles (below). We see that underestimates are greatest when the nearest road is between 500 and about 800 to 1000 meters away. This may indicate that the 500 meter cutoff for multiple road inclusion may be too small, e.g. if roads at 800 meters have an effect, then error would be introduced when only the nearest of several road segments located 600 meters away are included in the assessment.

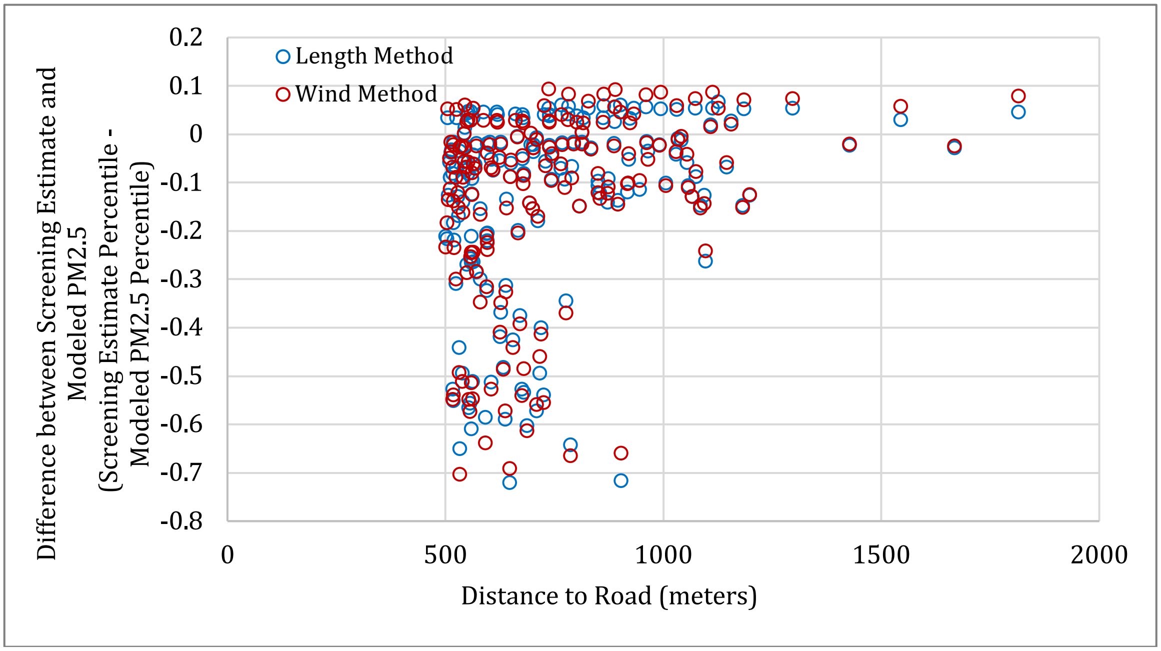

In the two figures below we similarly examine the 176 blocks in terms of the effective angle (angle between the block centroid and the road segment) and the traffic volume of the road segment. Note that effective angle of 1000 was used to signify calm conditions. There is no clear relationship to between angle or traffic volume and the ability of the Length and Wind method to match modeled PM2.5 percentiles.

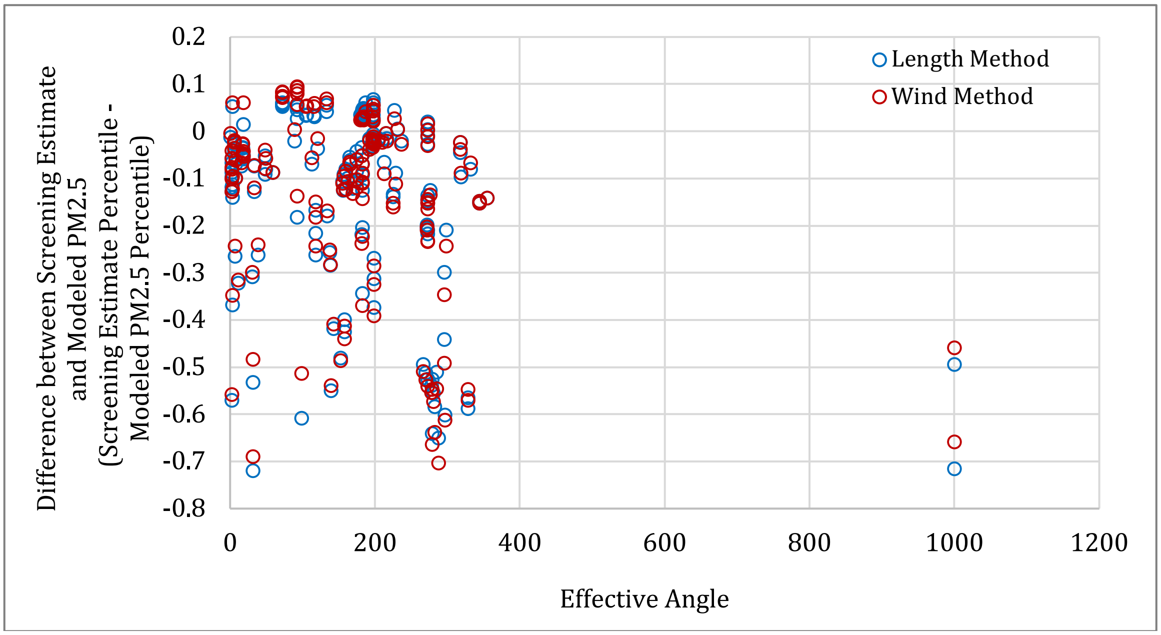
Difference between Screening Estimate and Modeled PM2.5 (Screening Estimate Percentile - Modeled PM2.5 Percentile)
0.2
0.1
0
-0.1
-0.2
-0.3
-0.4
-0.5
-0.6
-0.7
-0.8
0
200
400
600
800
1000
1200
Effective Angle
Length Method
Wind Method

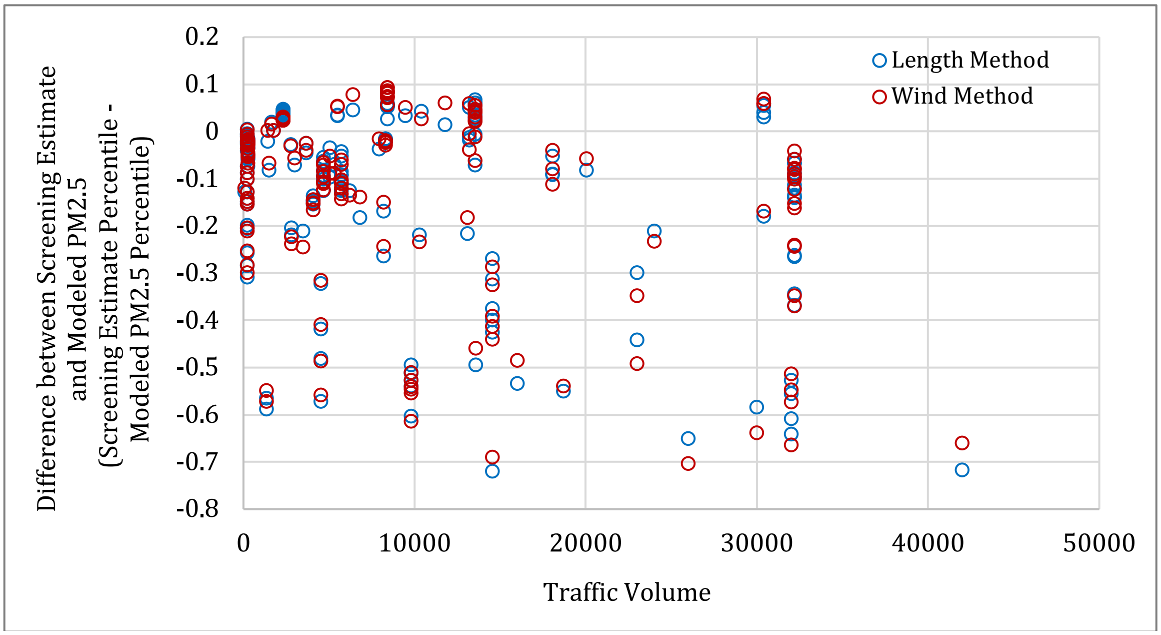
Difference between Screening Estimate and Modeled PM2.5 (Screening Estimate Percentile - Modeled PM2.5 Percentile)
0.2
0.1
0
-0.1
-0.2
-0.3
-0.4
-0.5
-0.6
-0.7
-0.8
0
10000
20000
30000
40000
50000
Traffic Volume
Length Method
Wind Method
